# Sidelobe Suppression Techniques for Near-Field Multistatic SAR

**DOI:** 10.3390/s23020732

**Published:** 2023-01-09

**Authors:** George A. J. Price, Chris Moate, Daniel Andre, Peter Yuen

**Affiliations:** 1 Radar & Electronic Warfare, QinetiQ, Malvern WR14 3PS, UK; 2 Centre for Electronic Warfare, Information and Cyber, Cranfield University, Defence Academy of the United Kingdom, Shrivenham SN6 8LA, UK

**Keywords:** SAR, multistatic, compressive sensing, UAS

## Abstract

Multirotor Unmanned Air Systems (UAS) represent a significant improvement in capability for Synthetic Aperture Radar (SAR) imaging when compared to traditional, fixed-wing, platforms. In particular, a swarm of UAS can generate significant measurement diversity through variation of spatial and frequency collections across an array of sensors. In such imaging schemes, the image formation step is challenging due to strong extended sidelobe; however, were this to be effectively managed, a dramatic increase in image quality is theoretically possible. Since 2015, QinetiQ have developed the RIBI system, which uses multiple UAS to perform short-range multistatic collections, and this requires novel near-field processing to mitigate the high sidelobes observed and form actionable imagery. This paper applies a number of algorithms to assess image reconstruction of simulated near-field multistatic SAR with an aim to suppress sidelobes observed in the RIBI system, investigating techniques including traditional SAR processing, regularised linear regression, compressive sensing. In these simulations presented, Elastic net, Orthogonal Matched Pursuit, and Iterative Hard Thresholding all show the ability to suppress sidelobes while preserving accuracy of scatterer RCS. This has also lead to a novel processing approach for reconstructing SAR images based on the observed Elastic net and Iterative Hard Thresholding performance, mitigating weaknesses to generate an improved combined approach. The relative strengths and weaknesses of the algorithms are discussed, as well as their application to more complex real-world imagery.

## 1. Introduction

### 1.1. Overview

As Unmanned Air Systems (UAS) develop, their applications in both civilian and military environments are rapidly diversifying. The ability to carry large sensing payloads presents an opportunity to integrate new sensors and imaging techniques, allowing for new capabilities in intelligence gathering. When applied to Radio Frequency (RF) imaging one of the key advantages of this over a traditional system—such as a Synthetic Aperture Radar (SAR) mounted on a fixed-wing aircraft—is the ability to produce highly multistatic images with different platforms sensing different parts of the RF spectrum or collecting signal from the scene at different aspect angles. Together with the potentially complex trajectories that can be flown, it is clear that there is a significant potential for high resolution and 3D imaging in such a system. However, the disjointed multistatic nature of the collection also presents one of the main image formation and processing challenges: suppressing sidelobes and other artefacts in the formed image due to the relative sparsity of the collected data. Through a number of research programmes, QinetiQ have developed a set of octocopters with RF imaging payloads to operate in this manner known as the RIBI system.

### 1.2. Previous Work

As a technique, SAR has been in use in military and civilian applications for over 60 years [1,2]. It is capable of producing 2D images with a long-range all-weather surveillance and reconnaissance capability, typically mounted on air or space platforms. Sidelobe suppression has been a common subject of research throughout its existence as the typical processing methods used are all imperfect recreations of the imaged scene. Recent research areas include receiver design [3], signal processing [4], and collection methodology [5]. As discussed multistatic SAR involves multiple sensing systems transmitting and/or receiving with techniques required to fuse these data into a single collection. In this process, there are a number of challenges such as clock accuracy [6] and geolocation accuracy [7].

Compressive Sensing (CS) [8,9] provides a technique for signal reconstruction under the assumption of sparsity in the signal, and allows for recovery even when this signal is sampled below the Nyquist Rate. It is applied in a number of fields including medical imaging [10,11,12], optical imaging [13,14], and radar [15,16,17].

For SAR, CS has been established as a method for image formation under specific conditions in previous work [18,19], and multi-static consideration has been given elsewhere in both active [20] and passive regimes [21]. In general, CS has shown promise as a technique for mitigating strong, extended, sidelobes in a number of challenging collection regimes:

- Background separation and Ground Moving Target Indication (GMTI) [22,23];
- Sparse multistatic collections;
- 3D imaging from a single pass (with complex trajectory) or few repeat passes [5,24];
- Gaps in frequency to avoid interference/jamming [25];

UAS have been considered before in work such as [20,26]; however, combining these into a single multistatic collection system with aperture synthesis between UAS presents new challenges. The Size, Weight, and Power (SWAP) requirements on a drone produce limitations on the realistic power that can be generated, and it has only recently become possible to provide a navigation and timing solution sufficient to allow collections from disparate UAS to be combined.

In the RIBI system [27], one of the key distinctions is the requirement for a near-field solution, and this is the focus of the work performed here. In a near-field regime a scatterer’s sidelobe structure will have a high dependence on position in the image, even for a single sensor. Combining data from multiple sensors leads to a challenging image to attempt recovery from; however, it can also generate additional scene information through diversity in spatial, frequency, and temporal variance [28]. This work specifically considers 2D imaging; however, with the accepted increase in processing power it is applicable to 3D volumetric SAR too.

In this work, nine algorithms will be evaluated from four broad areas of image formation. Initially a traditional SAR image formation algorithm, in this case back-projection, will be used as a baseline by taking a well-established technique in widespread use [29]. These algorithms are well-defined in a number of radar and SAR texts [30]. Windowing through use of Hanning or Hamming windows [31] is also applied as a separate case, as a simple addition to SAR processing already in use for controlling sidelobes in monostatic collections.

Unconstrained least squares approach the back-projection problem by looking to create pseudo-inverses of the measurement process, expressed in a matrix form. For this work, a simple least squares solution is applied as well as a pseudo-inverse using the Moore–Penrose Inverse [32].

Regularised linear regression techniques build upon least squares by adding in regularisation terms based on *l*-norms to bias the solution. Ridge [33] and Lasso [34] using $l_{2}$ and $l_{1}$ norms respectively on top of the least squares solution to select for limiting large coefficients or prefer a greater number of non-zero coefficients respectively. Elastic net [35] is a combination of Ridge and Lasso using a tuning parameter to combine the relative advantages of $l_{2}$ and $l_{1}$ norms into a single algorithm.

Finally, two explicitly sparse techniques in Orthogonal Matched Pursuit (OMP) [36] and Iterative Hard Thresholding (IHT) [37]. The referenced work on Iterative Hard Thresholding demonstrates that these techniques have already shown promise in adjacent SAR scenarios.

This set of algorithms was selected in order to give a sample across techniques that are either familiar to SAR already or have shown significant promise in literature elsewhere, whilst not necessarily being an exhaustive search of the entire space. This work will therefore apply these to the RIBI processing challenge to quantify their performance, and select for techniques that show the greatest benefit to a future near-field multistatic processing solution.

### 1.3. Contribution

This paper makes the following original contributions to the problem space:

- We investigate the potential of CS algorithms to address the currently unsolved issue of high sidelobes in multistatic SAR collections. These pose a challenge to SAR processing, as the sidelobes produced are capable of masking true scatterer returns and give the appearance of false returns in SAR images. Without this additional processing step, the quality of images produced is frequently unacceptable for human interpretability or further processing.
- We define quantitative metrics to determine the accuracy of two key aspects of SAR reconstruction: accuracy in reconstructing scatterers and effectiveness at suppressing sidelobe energy.
- We undertake a like-for-like comparison of the algorithms chosen on simulated 2D SAR data, utilising the quantitative metrics introduced earlier to down-select the algorithms for potential future application in multistatic SAR. Performance in terms of reconstruction accuracy and computational cost for each algorithm is assessed, as well as future real-world applicability and further development required. This allows a clear comparison of the algorithm performance for this radar application, and prioritises algorithms for others interested in multistatic SAR to consider.
- Finally we consider the potential of combining multiple existing algorithms into a single processing chain to achieve greater quantitative performance than their individual parts, as assessed by the metrics developed.

The paper will conclude with a discussion of the overall results of this research as well as a discussion about what necessary extensions would be required to process real data.

## 2. SAR Modelling

### 2.1. SAR Signal Model

The captured phase history $\Phi (f,t_{s})$ is a 2D array of amplitude and phase returns varying with transmitted frequency and slow time. A single phase change is given by:

(1) $$\phi =e^{-i2\pi f\tau }$$

where τ is the time delay between transmission and reception and *f* is the transmitted frequency. This can be rewritten to consider range instead:

(2) $$\phi_{k}=e^{-i\frac{2\pi f}{c}\left(R_{k}^{Tx}+R_{k}^{Rx}\right)}$$

where $R_{k}^{Tx}$ and $R_{k}^{Rx}$ correspond to the distance from the scatterer *k* to the transmitter and receiver respectively, and *c* is the speed of light. For a monostatic system generally $R_{Tx}\approx R_{Rx}$ or, if a common antenna is used, $R_{Tx}=R_{Rx}$. As the frequency modulates as a function of fast-time $t_{f}$ [30] and the radar moves in slow-time $t_{s}$ a total phase history, $\Phi_{k}$ can be constructed for this scatterer:

(3) $$\Phi_{k}=\zeta_{k}e^{-i\frac{2\pi f(t_{f})}{c}\left(R_{k}^{Tx}\left(t_{s}\right)+R_{k}^{Rx}\left(t_{s}\right)\right)}$$

which now includes a term to account for the scatterer Radar Cross-Section (RCS) $\zeta_{k}$. For a given scatterer this will be a complex value, however, modelling this can be challenging. This RCS can exist as a function of frequency, orientation, polarisation, and time, leading to some computationally inefficient simulations if all of these are accounted for. It is possible to use a matrix multiplication method as part of simulation design to accelerate this process, and typically this is what has been used where time is critical, while a second slower function (allowing for a more detailed simulation to be performed) is written separately.

Equation (3) can be simplified in the monostatic case to:

(4) $$\Phi_{k}=\zeta_{k}e^{-i\frac{2\pi f(t_{f})}{c}R_{k}\left(t_{s}\right)}$$

where $R_{k}\left(t_{s}\right)$ is the total path length:

(5) $$R_{k}\left(t_{s}\right)=R_{k}^{Tx}\left(t_{s}\right)+R_{k}^{Rx}\left(t_{s}\right)$$

The total phase history is the complex sum of all the individual scatterer contributions:

(6) $$\Phi_{total}=\sum_{k=1}^{k_{total}}\Phi_{k}$$

representing the data captured by the receiver due to the scene response. At present the precise nature of the waveform has not been considered. A linear chirp is used as a waveform, sweeping across the bandwidth as a discrete set of *N* frequencies $f_{1},f_{2},...,f_{N}$, with an aperture trajectory consisting of *M* collection points. This will result in an M×N phase history, which will be considered the basic data capture to be manipulated in all of the later algorithms.

The precise choices of variables such as the bandwidth, imaging geometry, and waveform, will have a significant and direct impact on the final image that can be formed. There are a set of relationships between these variables that will be of great significance when later image recovery algorithms explore image resolution and size.

(7) $$\delta \rho =\frac{\lambda_{c}}{2\Delta \theta }$$

(8) $$\Delta \rho =\frac{\lambda_{c}}{2\delta \theta }$$

(9) $$\delta R=\frac{c}{2B}$$

(10) $$\Delta R=\frac{c}{2\delta f}$$

**Figure 1:**
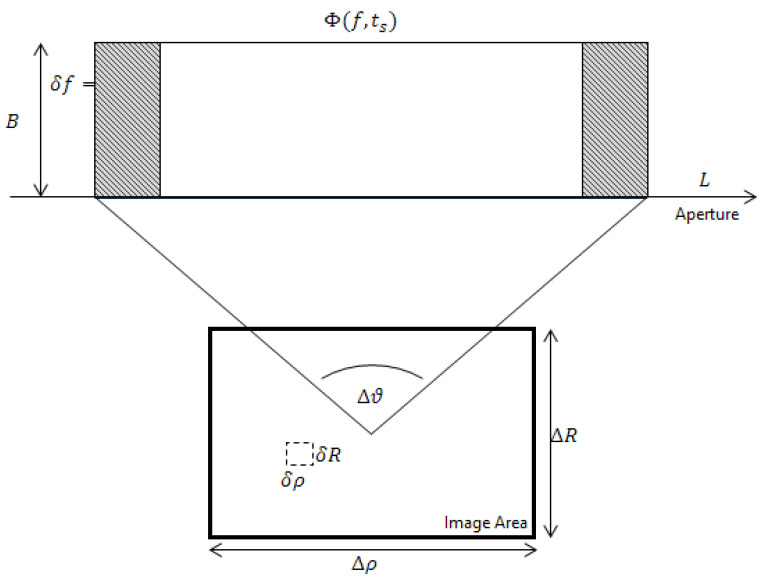
Comparing a full SAR aperture *L* of aperture angle Δθ with two smaller Nyquist-sampled apertures (grey areas) of bandwidth *B*, and attempting to reconstruct the full phase history Φ.

In these equations, *R* and ρ represent range and cross-range respectively, with δ being the finest resolution obtainable and Δ the maximum unambiguous image size. Thus the 2D image resolution (δ) and ambiguity (Δ) limits can be fully defined by the Equations (7)–(10). These can be seen on Figure 1.

### 2.2. Image Formation

With RIBI as an example collection scenario, the algorithms selected will be applied to the multistatic SAR case. The intention is to address the strong sidelobes observed when combining multistatic collections. Whilst the RIBI system is capable of collecting in different frequency bands, here only gaps in azimuth coverage are considered; however, the techniques investigated could equally be extended to cover gaps in frequency. In this case, the strong sidelobes would occur in range as opposed to cross-range.

Equations (7)–(10) set out relationships for resolution limits assuming a grazing angle of zero degrees. The cross-range resolution, δρ, of a SAR image is dependent upon the aperture angle in azimuth, which for a monostatic collection extends for the entire aperture *L*. In the multistatic case processed as part of this paper, a gap consisting of zeros is created in a monostatic aperture, leaving two smaller sub-apertures on each end of the collection, which will be referred to as a pseudo-multistatic aperture. Now that the aperture angle for each sub-aperture has significantly decreased, the resolution limit in cross-range is significantly degraded; however, as the bandwidth, *B*, and frequency spacing, δf, have not been affected there will be no loss of range resolution. In addition to this, there will be strong sidelobes due to the changed geometry and thus shape of k-space support. It would be expected that these will lead to extended curves centred on the two small apertures. The aim of this work is therefore to improve final image resolution by assuming that smaller multistatic collections are part of one larger collection, achieving the resolution of a comparable full monostatic aperture.

This requires a significant amount of data to be reconstructed that is missing from the collection. From using the poor-resolution images, with the associated knowledge of time and position, it will be possible to generate a scene estimate, simulate the expected response, and iterate the scene estimate to determine the positions of scatterers.

Whilst SAR processing is traditionally performed via signal processing functions this work makes use of a matrix-based approach. A SAR collection can be described in terms of a matrix transformation:

(11) Φ=AΓ+n

which defines Φ as the complex phase history collected for measurement scene Γ through use of a ’measurement matrix’ *A*. Γ can be considered to be a map of reflectivity through a region of space to be imaged. Noise *n* is present as a random phase addition to each term of Φ. The phase history represents the data captured by the receiver, which is the sum of the returns from all *K* scatterers in a scene Γ:

(12) $$\Phi =\sum_{k=1}^{K}\zeta_{k}e^{-i\frac{4\pi f\left(t_{f}\right)}{c}R_{k}\left(t_{s}\right)}$$

where frequency *f* varies in fast-time and the path length to scatterer $k,d_{k}$, varies in slow-time. $a_{k}$ is the complex RCS of a scatterer which is assumed to not vary with frequency or angle. A collection of scatterers in free space can therefore be defined and the total response from the scene calculated. This phase history simulation shall be referred to as ’forward-projection’. In order to use a matrix approach, the scene is assumed to consist of a two-dimensional grid of (x,y) coordinates with each coordinate having an associated complex RCS (height can also be included but has not been in the current work). Thus Γ can be defined as an $\left(N_{x}N_{y}\times 1\right)$ matrix, and the phase history can be represented in this manner as an $\left(N_{f}N_{t}\times 1\right)$ vector:

(13) $$\Gamma =\left[\begin{array}{c} a\left(x_{1},y_{1}\right)\\ a\left(x_{2},y_{1}\right)\\ \vdots \\ a\left(x_{N_{x}},y_{1}\right)\\ \vdots \\ a\left(x_{N_{x}},y_{N_{Y}}\right) \end{array}\right],\Phi =\left[\begin{array}{c} \varphi \left(f_{1},t_{1}\right)\\ \varphi \left(f_{2},t_{1}\right)\\ \vdots \\ \varphi \left(f_{N_{f}},t_{1}\right)\\ \vdots \\ \varphi \left(f_{N_{f}},t_{N_{t}}\right) \end{array}\right]$$

In these, we have used $N_{f},N_{t},N_{x},N_{y}$ to describe the number of frequency (fast-time), slow-time, *x* coordinates, and *y* coordinates respectively. There is also an assumption that the RCS does not vary with frequency or angles of incidence/reflection. Hence *A* is an $\left(N_{f}N_{t}\times N_{x}N_{y}\right)$ matrix that contains the phase change terms:

(14) $$A=\left[\begin{array}{ccc} e^{-i\frac{4\pi f_{1}}{c}R_{k}\left(t_{1},x_{1},y_{1}\right)} & \ldots & e^{-i\frac{4\pi f_{1}}{c}R_{k}\left(t_{1},x_{N_{x}},y_{N_{y}}\right)}\\ \vdots & \ddots & \vdots \\ e^{-i\frac{4\pi f_{N_{f}}}{c}R_{k}\left(t_{N_{t}},x_{1},y_{1}\right)} & \ldots & e^{-i\frac{4\pi f_{N_{f}}}{c}R_{k}\left(t_{N_{t}},x_{N_{x}},y_{N_{y}}\right)} \end{array}\right]$$

Here the path length has been rewritten as a function of slow-time and the scene (x,y) coordinates. This path length refers to the three-dimensional distance from transmitter, to the scene coordinate, and back to the receiver. This then gives the matrix representation of a SAR collection, with Φ representing the data collected by receiver.

Creating an image will be dependent on creating a representation of *A*, generally referred to as $A^{\prime }$, to pre-multiply Φ by to create images $\Gamma^{\prime }$. These images will be estimations of the reflectivity map Γ, with any errors introduced by transformations between *A* to $A^{\prime }$ present as sidelobes.

(15) $$\Gamma^{\prime }=A^{\prime }\Phi$$

**Figure 2:**
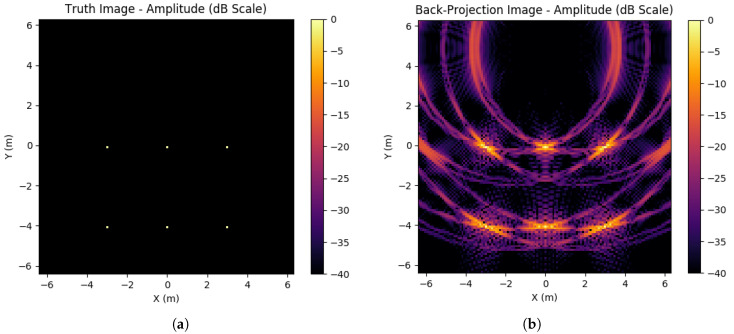
Ground truth and corresponding back-projection image. This back-projection image is formed from the absolute value of the complex image data. (**a**) Ground truth of 6 scatterers of RCS 1; (**b**) Back-projection image ($A^{T}\Phi$) from an aperture of 10% data available.

In the ideal case, an exact inversion of *A*, $A^{-1}$, would exist such that the ground truth estimation generation would exactly match the ground truth used. However, in general $A^{-1}$ will not exist, and any noise will render this approach useless. Therefore, other methods of estimating $A^{\prime }$ as a general solution to Equation (15) should be used. These alternative methods will generate artefacts in the image in the form of sidelobe artefacts around each scatterer. An example of this imperfect inversion can be found in Figure 2, showing how sidelobes will form. Due to being near-field the sidelobe structure will vary significantly across the imaging space, whereas a far-field regime would expect approximately equivalent sidelobes for all scatterers.

### 2.3. Research Objective

The objective of this work is to find methods for estimating $A^{-1}$ such that:

- Scatterer RCS is preserved;
- Excess energy is effectively suppressed;
- Computational cost to achieve the above is not excessive;

Excess energy is defined as any pixels of the reflectivity map that contain energy that is not directly attributed to a scatterer within the ground truth. As such, it measures the magnitude of any sidelobes present at the conclusion of the image formation process.

The following sections will consider different methods for performing this reflectivity map estimation in Equation (15). Initially traditional SAR-like techniques will be considered as a baseline, before moving onto linear regression and CS techniques.

### 2.4. Experimental Design

In order to test the algorithms selected, a simulation based on the signal model described in Section 2.1 has been developed. This uses the phase changes along the path lengths described and summation across a series of scatterers to generate a phase history. The scatterers are modelled as infinitesimally small points with uniform RCS. The scatterer positions, radar parameters, and aperture geometry are designed to replicate real data collected using Cranfield University’s Ground-Based SAR GBSAR [38] system for future work.

A simulated image is formed from six of these scatterers arranged in a regular 3 × 2 grid in free space. From this Φ was simulated using Equation (6) to represent the data collected at the receiver. From this a series of modifications can be made to Φ in order to test various properties of the algorithms. Firstly, pulses can be removed in order to create the pseudo-multistatic apertures required, with varying percentages of data removed. Secondly, Gaussian noise can be added to test how the algorithms will respond to non-ideal cases. This Gaussian noise is added to the real and imaginary components of the signal independently and then combined.

In order to assess algorithm performance, two approaches have been taken. The first is a simple visual inspection of the reproduced ground truth, in order to assess the human interpretability of the images. As there is no quantitative metric associated with human interpretability, two numerical methods have been applied based on Mean Squared Error (MSE):

(16) $$\mathrm{MSE}=\frac{1}{n}\sum_{i=1}^{n}{\left(Y_{i}-{\hat{Y}}_{i}\right)}^{2}$$

where $Y_{i}$ and ${\hat{Y}}_{i}$ are two vectors to be compared with *n* total elements. The MSE is computed for two sections of the image independently, operating in each case on Γ and $\Gamma^{\prime }$.

The first of these is to examine the positions that the six scatterers should be at and compute the MSE between these six amplitude values in the ground truth and the estimated ground truth. This is to assess whether the algorithm has successfully located the expected scatterers and scaled their amplitudes appropriately.

The second of these is to compute the MSE of the rest of the pixels with the scatterers excluded as a measure of excess energy in the image. This is to assess the algorithms’ abilities to suppress strong sidelobes that are observed in multistatic images.

## 3. Algorithm Downselection

### 3.1. Back-Projection

The first techniques to be considered come from traditional SAR image formation methods. The matrix approach used in this work is not necessarily typical of radar processing; however, the multiplication steps work in the same fashion as the looping of a functional back-projection algorithm [30].

Back-projection within this matrix scheme can be approximated by pre-multiplying the phase history by the transpose of the A-matrix:

(17) $$\Gamma_{BP}=A^{T}\Phi$$

This will inevitably lead to sidelobes forming in the ground truth estimation due to the difference between the transpose and inverse matrices.

A standard technique for managing sidelobes involves applying windowing functions, which will mitigate the sinc-like ringing effects of the Fourier Transforms that are being performed on the collection when forming images. These can take the form of Hamming or Hanning weightings applied in range and/or cross-range.

(18) $$\Gamma_{BP}={A_{W}}^{T}\Phi$$

**Figure 3:**
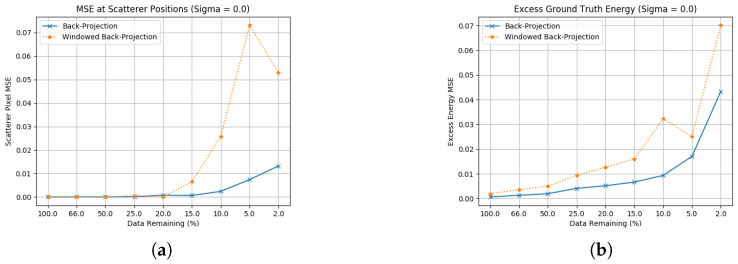
Ground truth estimation statistics for traditional SAR methods. (**a**) Scatterer location RCS error without noise; (**b**) Excess energy without noise.

Figure 3 shows MSE results for back-projection and windowed back-projectionn, with Figure 3a showing how accurate the point scatterer RCS is estimated and Figure 3b showing energy present in the reflectivity map that is in excess of the simulated scatterers. These results show that while existing SAR techniques may function well for traditional apertures they are insufficient for the multistatic imaging scheme presented; however, back-projection still serves as a useful comparison in later sections. Back-projection alone is not capable of achieving the correct scatterer amplitudes for highly-reduced apertures and also introduces significant excess energy in the forms of large, extended sidelobes. The use of windowing functions is insufficient to manage these sidelobes, and is shown to worsen performance against these metrics. There is also an increased instability in the quantitative performance due to interference effects between the two sections of aperture, which is shown in the windowed case for 5% of the data remaining. Given that these techniques represent SAR, it is necessary to incorporate different methods outside the traditional radar domain for reflectivity map estimation. In the following sections (Section 3.2, Section 3.3 and Section 3.4), three groups of these are considered, with back-projection used as a baseline for judging improvement.

### 3.2. Unconstrained Least Squares

The first non-SAR approaches applied are two least squares approaches with no regularisation terms applied: these are least squares and the Moore–Penrose inverse.

The least squares approach taken will apply the following to the SAR equation:

(19) $$\min_{\Gamma }{∥\Phi -A\Gamma ∥}_{2}^{2}$$

Pseudo-inverse algorithms use techniques such as the Moore–Penrose inverse [32,39] to create an estimate of $A^{-1}$, $A^{+}$, as in the following:

(20) $$\Gamma_{PI}=A^{+}\Phi$$

The implementation uses singular-value decomposition to compute a generalised inverse of *A*.

**Figure 4:**
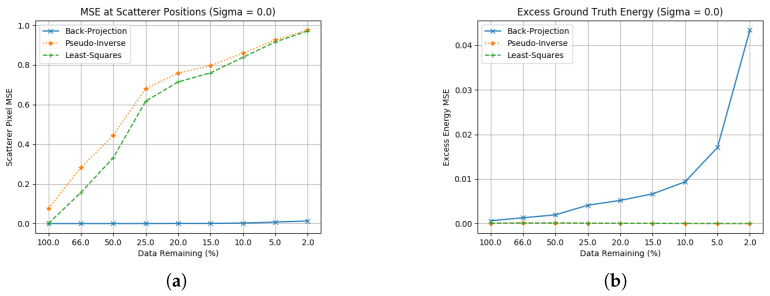
Ground truth estimation statistics for unconstrained least squares methods. (**a**) Scatterer location RCS error without noise; (**b**) Excess energy without noise.

As per Figure 4, these two methods are not seen as effective in this imaging scenario. Both techniques are capable of identifying the scatterer positions; however, they do not accurately scale the RCS, with performance degrading as the data are removed from the aperture. They do effectively suppress excess energy compared to back-projection, although with low peak RCS at the scatterer positions this low excess energy is due to low amplitude across the whole image. Two additional drawbacks of these techniques are their low computational efficiency and lack of resilience to noise. The significant computational cost of attempting these inversion techniques significantly constrains the image size that can be formed in a reasonable time, and even the smallest amount of Gaussian noise added will cause large inaccuracies in reconstruction across the whole image. These techniques need improvement through regularisation terms, which is the focus of the next section.

### 3.3. Regularised Linear Regression

In order to constrain least squares, a regularised approach is preferred. Three representative algorithms have been considered in this section using linear regression to solve for $\Gamma^{\prime }$. These are Ridge [33,40], Lasso [34,41], and Elastic net [35].

Ridge attempts to mitigate the issues observed in Section 3.2 by introducing a penalty term to suppress large values present in estimates of Γ using the following equation:

(21) $$\min_{\Gamma }{∥\Phi -A\Gamma ∥}_{2}^{2}+\alpha {∥\Gamma ∥}_{2}^{2}$$

In Lasso, the $l_{2}$ penalty term is replaced with an $l_{1}$ penalty, and is more suited to sparse imaging as in the case presented. The $l_{1}$ norm will prefer a solution with fewer non-zero elements in it, with a tuning parameter α available to select how strictly this should be applied. This penalty term is applied to the least squares solution as follows:

(22) $$\min_{\Gamma }{∥\Phi -A\Gamma ∥}_{2}^{2}+\alpha {∥\Gamma ∥}_{1}$$

Finally, Elastic net seeks to combine regularisation of $l_{2}$ and $l_{1}$ norms as shown in Equation (23). The tuning parameter α is present as in Lasso and Ridge; however, now a second parameter β is available to balance between $l_{1}$ and $l_{2}$ norms-effectively preferring qualities of Lasso or Ridge as required. This enables Elastic net to benefit from the stability of Ridge whilst also selecting for sparse solutions as in Lasso.

(23) $$\min_{\Gamma }\frac{1}{2N_{samples}}{∥\Phi -A\Gamma ∥}_{2}^{2}+{\alpha \beta ∥\Gamma ∥}_{1}+\frac{\alpha (1-\beta )}{2}{∥\Gamma ∥}_{2}^{2}$$

**Figure 5:**
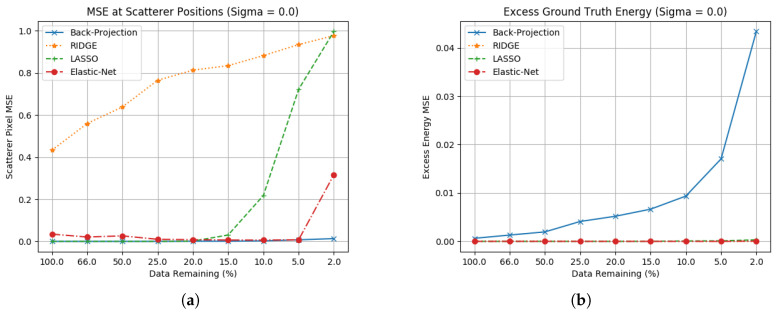
Ground truth estimation statistics for regularised linear regression methods. (**a**) Scatterer location RCS without noise; (**b**) Excess energy without noise.

**Figure 6:**
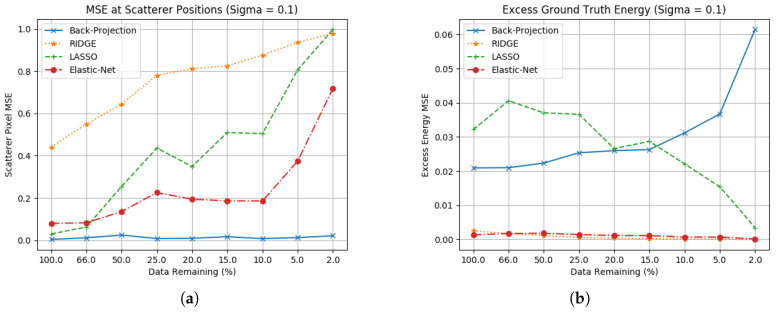
Ground truth estimation statistics for regularised linear regression methods with noise. (**a**) Scatterer location RCS error including noise (σ=0.1); (**b**) Excess energy including noise (σ=0.1).

For each of these methods, the parameters α and β were optimised through use of a specified training image. The MSE results for these three algorithms compared to back-projection are found in Figure 5 and Figure 6. For these results, two sets of data have been included. Figure 5a,b shows MSE results for images formed without noise, and Figure 6a,b showing datasets where Gaussian noise (σ=0.1) is added to the phase history.

- Ridge is an improvement on Pseudo-Inverse techniques only in speed, but is still computationally inefficient. While it correctly identifies scatterer positions and suppresses excess energy it does not scale scatterer amplitudes correctly. During parameter optimisation, it was found that in order to effectively suppress excess energy the $l_{2}$ norm also suppressed the scatterer amplitudes.
- Whilst Lasso performs better in both scatterer amplitude estimation and excess energy suppression, this is limited to collections with larger apertures and in the absence of noise. Apertures with a significant amount of energy subtracted will lead to poor reconstruction, and while it can suppress sidelobes in the absence of noise this also breaks down quickly. Computational cost is also significant.
- Elastic net is the best of these three methods, with strong performance on both scatterer estimation and excess energy suppression. If parameters are selected effectively, this realises the benefits of Ridge’s energy suppression whilst engaging Lasso’s advantage in generating sparse results.

These techniques show that linear regression can be used in this imaging regime; however, there are still challenges with regards to the long computation times and vulnerability to noise.

### 3.4. Hard-Limited Algorithms

The final set of algorithms considered were two greedy algorithms with hard limits on the number of non-zero coefficients allowed in $\Gamma^{\prime }$. These are OMP [21,42] and IHT [43]. In each of these algorithms the number of non-zero coefficients will be denoted by as $N_{>0}$.

OMP minimises the $l_{2}$ norm whilst restricting the number of non-zero coefficients of Γ via [36]:

(24) $$\min_{\Gamma }{∥\Phi -A\Gamma ∥}_{2}\;\mathrm{subject}\;\mathrm{to}\;{∥\Gamma ∥}_{0}\leq N_{>0}$$

IHT imposes sparsity on the ground truth estimate by applying a hard threshold $H_{s}$ to the coefficients, iterating until the error between $\Gamma^{\prime }$ and Γ is suitably low through [37]:

(25) $$\Gamma_{n+1}=H_{s}\left(\Gamma_{n}+A^{T}\left(\Phi -A\Gamma_{n}\right)\right)$$

**Figure 7:**
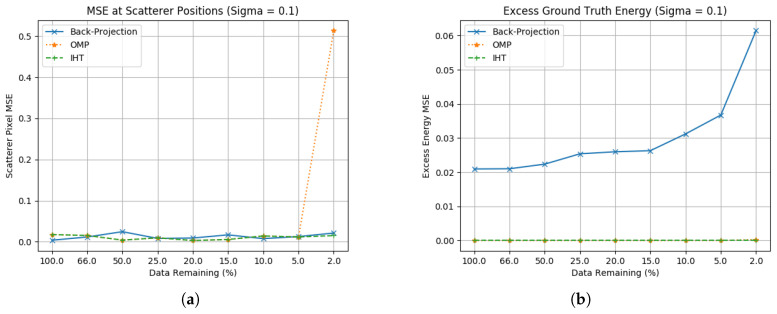
Ground truth estimation statistics for hard-limited CS methods. (**a**) Scatterer location RCS error including noise (σ=0.1); (**b**) Excess energy including noise (σ=0.1).

As both of these algorithms have explicit limits on the number of non-zero elements that they allow—and not coefficients such as α in Section 3.3—they can be optimised to the exact number of scatterers expected in these ground truths. The wider validity of this approach to images without a known ground truth is discussed during Section 3.5. The MSE results for these plots are contained in Figure 7.

Figure 7a,b show results with Gaussian noise added. While performance in the absence of noise is trivial for these algorithms, both of these techniques have similar performance in that they have extremely low scatterer error while they are able to identify the scatterer positions effectively. At the point where only two range profiles are added and any intersection of sidelobe patterns become viable as a scattering position, and thus performance breaks down. Low excess energy is expected due to the explicit limit on the number on non-zero elements in $\Gamma^{\prime }$ that are permitted. In this case $N_{>0}=6$ with precisely six scatterers; therefore, if the six scatterers are successfully located there are no sidelobe pixels permitted in the solution, and thus no sidelobes are present. Whilst this represents strong performance there are two significant caveats. The first is that these techniques require an accurate estimate of $N_{>0}$ to perform effectively, and a real image will not have this information available. The second issue is whether a real-world image can be considered sparse enough to apply this hard limit. An airborne image of terrain would consist of pixels that almost all contain information and therefore attempting to set a value for $N_{>0}$ would risk removing energy. One area of interest for future research would be to look at techniques, such as coherent change detection, for generating images with fewer pixels of interest that may be more suitable.

### 3.5. Analysis

**Figure 8:**
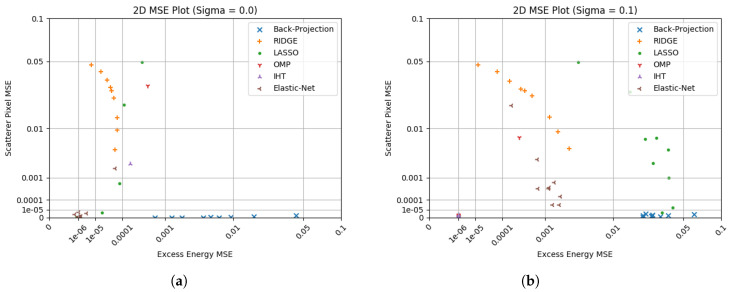
Analysis of 5 algorithms compared to back-projection via logarithmic 2D plotting. Note that any exactly 0 values have been defaulted to $1\times 10^{-5}$ to assist log plotting. (**a**) 2D scatterer vs. excess energy plot without noise; (**b**) 2D scatterer vs. excess energy plot including noise (σ=0.1).

For overall analysis a number of the algorithms have been discounted based on their performance in the preceding sections:

- Windowed back-projection: was not capable of producing a significant improvement over back-projection;
- Pseudo-inverse: lacked any resilience to noise to form meaningful results;
- Linear regression: also lacked resilience to noise, and 3 derivative algorithms are already present which improve on this technique;

Figure 8 shows 2D plotting of results in previous sections in order to look at grouping algorithm performance. In these the results for each algorithm are given for varying sparsities at one specific noise value, with Figure 8a showing results in the absence of noise and Figure 8b showing results with noise added.

Comparing the regularised linear regression techniques shows that Elastic net is an improvement on both Ridge and Lasso. Lasso is able to correctly identify and scale scatterers but at the cost of significant excess energy, whilst Ridge’s preference to suppress overall amplitudes means it is unable to scale the RCS of scatterers effectively. Elastic net shows that it is able to effectively manage both of these, outperform them at either role, and shows strong tolerance to noise and aperture reduction. Of these, Elastic net represents an effective blend of the two techniques providing its parameters can be set appropriately.

Both OMP and IHT function effectively with the significant caveat of requiring an accurate estimation of $N_{>0}$ to function as discussed previously. Their hard limits on sparsity mean that unless they miss a scatterer position their excess energy is almost certainly low, and given a favourable value of $N_{>0}$ they are able to effectively reconstruct scatterers. As they both appear to be effective and do not introduce significant processing cost, they are valuable to consider in additional work. As discussed in Section 3.4, there are potential issues around applicability to non-sparse real data.

At this point, Ridge and Lasso are discounted from further simulation due to Elastic net’s ability to combine both of their strengths without accepting the compromises required for their use. Therefore later work will comprise comparisons between traditional back-projection, Elastic net, OMP, and IHT.

## 4. Additional Simulations

In order to assess the performance of the algorithms on a more diverse set of data, an additional dataset is presented as part of this work.

### 50 Scatterers

**Figure 9:**
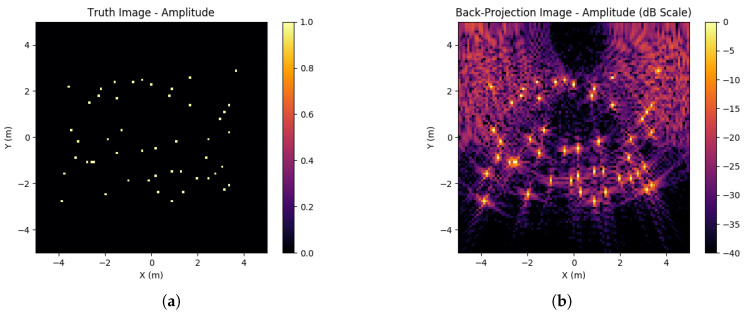
Ground truth and corresponding back-projection for 50 randomly-placed scatterers. (**a**) Ground truth of 50 randomly placed scatterers; (**b**) Image formed via back-projection for full aperture and no noise.

**Figure 10:**
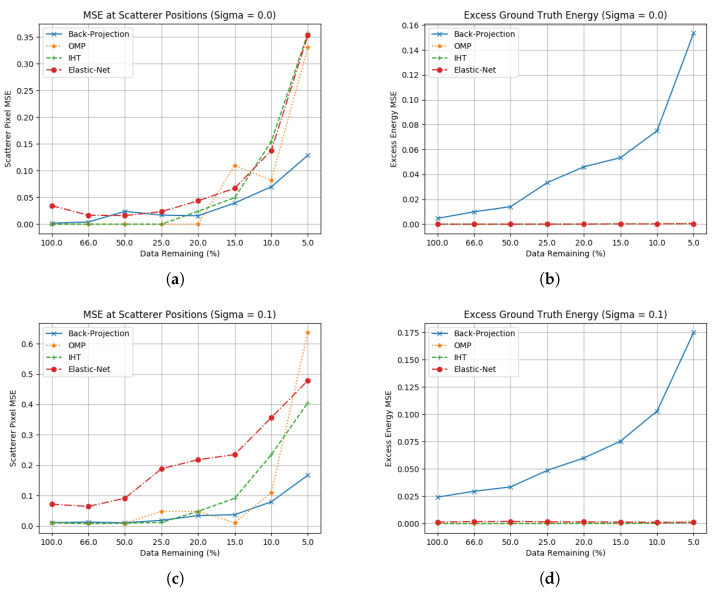
Ground truth estimation statistics for 50 randomly-placed scatterers. (**a**) Scatterer location RCS error without noise; (**b**) Excess energy without noise; (**c**) Scatterer location RCS error including noise (σ=0.1); (**d**) Excess energy including noise (σ=0.1).

In this scene, 50 scatterers are placed randomly within the imaging space, representing 0.5% of the pixels containing RCS to reconstruct. These are all of constant RCS=1. It is clear that OMP and IHT will be unable to effectively recover this scene with $N_{>0}=6$; therefore, this has been raised to $N_{>0}=50$ to match the number of scatterers. Elastic net has been left with the same parameters as has been used for the previous scene. Example ground truth and SAR image for this are shown in Figure 9 and the results of this are shown in Figure 10.

The results show that all three algorithms are still able to effectively suppress the sidelobes generated; however, their ability to estimate scatterer position and RCS is not as clear as previous, more simple cases. Here the complex sidelobe structures overlap scatterers more frequently, making it more challenging to distinguish excess energy from scatterers.

It is worth noting that at this point the optimisation methodology has diverged for the different techniques. Whilst OMP and IHT have been allowed to be re-optimised for this case, this was not done for Elastic net. These results suggest that Elastic net may be more widely applicable to scenes where the ground truth is not known.

## 5. Further Algorithm Development

Following the downselection and tests on a randomised dataset, an approach was taken to combine multiple algorithms into a single processing function, considering the respective strengths shown by the algorithms. Elastic net had proved effective at identifying scatterer locations but not necessarily estimating their RCS in the presence of noise. In comparison, OMP and IHT were effective at both noise suppression and scatterer estimation but were dependent on an estimation of the number of scatterers in the scene.

**Figure 11:**
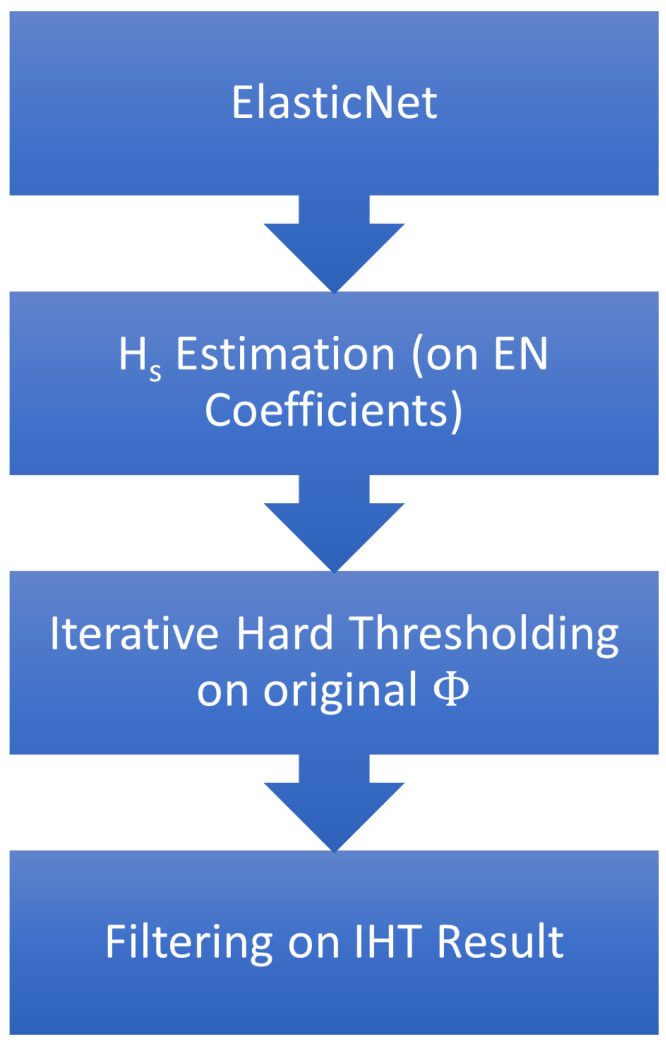
Novel processing chain for ground truth estimation in sparse SAR images.

**Figure 12:**
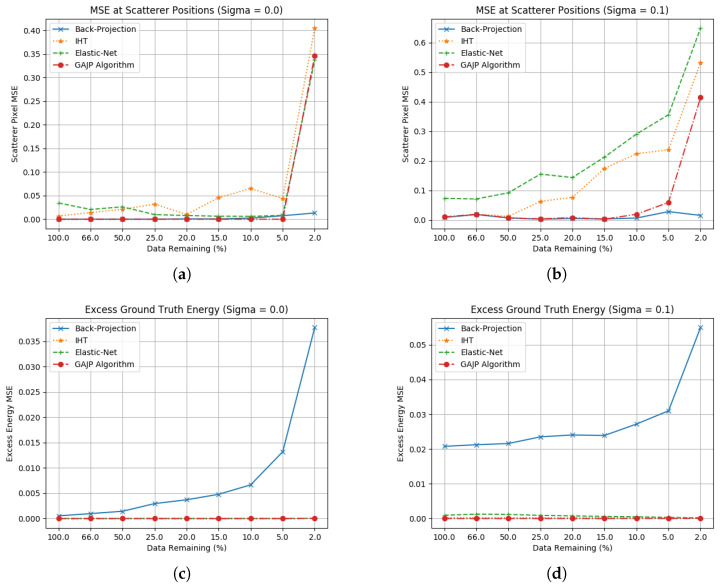
Ground truth estimation statistics for the new processing chain against 20 randomly-placed scatterers. (**a**) Scatterer location RCS error without noise; (**b**) Excess energy without noise; (**c**) Scatterer location RCS error including noise (σ=0.1); (**d**) Excess energy including noise (σ=0.1).

The final processing chain (as per Figure 11) used in this work used Elastic net as the first stage in the processing to recover an approximate number of scatterers, with an estimate of the number of scatterers being generated by counting pixels down −10 dB of the maximum value in the image. This was passed to IHT as its estimate of $H_{s}$ to use, as well as a mask of where Elastic net placed the scatterers with an expanded search area around each pixel. The results of this algorithm are in Figure 12, calculated against 20 random scatterers. In this case, the IHT comparison is given 100 scatterers (as 1% of the image) so it is not specifically optimised for this imaging scenario. If IHT is passed the correct number of scatterers, it should achieve the same solution as the novel algorithm; however, the plots consider how this processing chain would perform compared to blind use of a poorly-optimised IHT algorithm.

As a preliminary attempt at creating a more general approach, this shows that there is potential in the use of Elastic net as an estimation tool for setting the number of non-zero coefficients in an IHT solution. Whilst Elastic net still needs parameters estimating, it may be possible to achieve this elsewhere (e.g., use of image statistics to determine Elastic net learning rates) and as such make it a more attractive algorithm to attempt to optimise than IHT. This will be especially relevant in attempting to apply these algorithms to real data, where an estimate of $H_{s}$ will be difficult to generate in advance.

## 6. Analysis

### 6.1. Simulated Results

Over the two sets of results presented, a number of key outcomes have emerged.

Existing SAR techniques are not suitable for multistatic apertures with significant spatial gaps in collections or where those collections are small. The loss in cross-range resolution is too great to overcome and sidelobe interference effects mean that sidelobes are too strong to produce images with any interpretability. In addition, simple least squares or pseudo-inverse approaches to solve the SAR linear equation are insufficient due to the presence of noise in the collected signal. In order to achieve viable reconstructed images either regularised linear regression or intentionally sparse processing methods can generate images that reconstruct the ground truth successfully.

Of these approaches, three have been shown to have the most effective performance when assessed by quantitative image metrics. These are OMP, IHT, and Elastic net, and their overall performance is assessed below.

OMP and IHT are both effective algorithms for suppressing sidelobes and recovering scatterers. Their parameter optimisation is, however, a significant concern when it comes to wider application. In all of the datasets analysed, it has been assumed that the ground truth is known to an extremely high precision, which for a general SAR collection is not true. Even then, bothrequired changing to be suitable for the 50 scatterer dataset compared to six scatterers, and this high bias is another significant drawback. The MSE-based metrics used also rely on having a ground truth to compare the images against; therefore, to handle real images, either an alternative methodology for image quality assessment would be required or an accurate ground truth would need to be estimated/generated.

Elastic net has shown significant promise and whilst it has α and β parameters to select these did not require modification across these three datasets. It is still likely that this would require optimising for a given radar system, scene, or geometry; however, it may have a wider applicability than OMP and IHT if the tuning parameters are sufficiently stable. If further research showed that these parameters were not too sensitive, and unlikely to introduce significant bias, this would represent a route to future implementation of these approaches in multistatic SAR images.

Of the three algorithms investigated throughout the work, Elastic net appears to function the best based on the criteria outlined throughout this paper. It was able to accurately reconstruct scatterers (albeit with degraded performance against noise) whilst simultaneously effectively suppressing sidelobes and noise. It did not require the same precise information about the numbers of non-zero elements to allow and, once optimised, performed well across all three datasets presented. In future this could be expanded to investigate how effectively it can be optimised across more datasets and whether this can then be successfully applied to real images. Cross-validation methods could be used on a much larger training dataset to optimise α and β, and work done to see how generalised this can be made in terms of geometry, scenes, radar parameters, and noise.

Expanding beyond the original algorithms and into novel algorithms as per Section 5 shows that the relative strengths of these algorithms can be combined to achieve better performance than the sum of their parts. As noted, the strength of IHT/OMP in being able to suppress noise and recover scatterers is highly dependent on an accurate estimate of the number of non-zero coefficients; however, Elastic net could yield an approach for estimating this figure.

### 6.2. Expansion to Real Imagery

All of the work considered here has been applied to simulated data, so the following section considers what extensions would be required in order to achieve performance in real collections.

Firstly, while Equation (11) can capture any given collection process, it has only been used with an *A* matrix that considers perfect point scatterers. A real scatterer would be assumed to have variations in RCS as functions of frequency, azimuth and elevation angles, and polarisation. The previous model for scatterering where $\zeta_{k}$ (Equation (6)) was invariant would now be written in terms of:

(26) $$\zeta \left(f,\theta ,\psi ,P\right)e^{-i\frac{4\pi f}{c}R_{k}\left(t,x,y\right)}$$

where ζ now encodes all of these additional RCS variations as functions of frequency, azimuth, elevation, and polarisation respectively. As different scatterers will have different RCS models a number of different ζ could be hypothesized, with corresponding *A* matrix constructions to apply. For example, were Γ to be the positions of a number of trihedrals an estimation of $\zeta_{\left(trihedral\right)}$ could be obtained to apply to the elements of *A*. If ζ is used as a catch-all term, it can also encode other sources of imperfect behaviour such as receiver characteristics and, atmospheric effects (likely negligible at short-range but vital at longer ranges); however, the trade-off here is a more complex *A* matrix to compute and successfully estimate the inverse. Different types of scatterers will have different ζ models, meaning *A* will need to be able to encode a number of different scatterers independently, which will significantly increase memory requirements.

Motion compensation is also a vital part of SAR imaging, and until this point it has been assumed that a perfect motion solution has been obtained. For a multistatic UAS-based system, there are not only imperfections in GPS/IMU data to consider but also errors between the individual clocks that each platform will be running on attempting to combine data from multiple sensors. In general, errors in the motion solution will lead to defocusing effects in the image and thus a deviation from the perfect motion/perfect scatterer model used here. If the SAR linear equation is unable to account for this defocusing, it is highly probable that these algorithms will struggle to successfuly reconstruct the image; therefore, some attempt to include a model for motion error and subsequent defocusing would be beneficial to include in *A*. That said, this may prove to be difficult to estimate, and as such, effort spent improving the motion solution itself would likely yield better results.

The assessment of performance used for previous sections will also no longer be applicable. In these simulated data, the ground truth is well-defined and controlled. This will present issues both in optimisation of algorithms and in assessment of performance; however, alternative methods and metrics such as signal-noise ratio, contrast, or skewness could be utilised in this case.

## 7. Conclusions

### 7.1. Main Results

The work presented has shown traditional radar image formation algorithms to be insufficient at suppressing sidelobes in the near-field multistatic imaging case relevant to RIBI, and therefore alternative techniques are required. As a solution to this problem, regularised linear regression and CS techniques can be used for sidelobe suppression in simulated SAR imagery. This work has been quantitatively assessed against two datasets to downselect the most effective algorithms in this case, considering the accuracy of scatterer reconstruction, suppression of excess energy, and computational cost. Based on this assessment methodology, Elastic net, OMP, and IHT are all demonstrated as capable of suppressing excess energy whilst preserving scatterer reconstruction accuracy. Least squares techniques lacking the regularisation terms do not have sufficient robustness to noise to produce effective reconstructions, and Elastic net is an improvement on both Ridge and Lasso techniques.

It has also been shown through a novel processing chain that their relative strengths can be utilised to produce results that improve upon their individual performance. For a practical image formation algorithm; however, there are challenges remaining around parameter selection, real-world ground truthing, and computational cost.

### 7.2. Future Work

Future work has two main strands. The first of these is to attempt to refine the algorithm proposed in Section 5 by exposing it to more varied simulated data. Of particular relevance will be to attempt to set Elastic net parameters based on statistics of the SAR images such as SNR. In this way, a processor can be developed which is agnostic of input data and requires little training once the parameter estimation methodology is fixed. The second key area of work is to expand this to real data, by performing an in-depth analysis of how real scattering primitives appear in SAR images and what expansion of *A* is required to model this.

## Data Availability

The data are not publicly available due to legal considerations.
